# The Scent of Stress: Evidence From the Unique Fragrance of Agarwood

**DOI:** 10.3389/fpls.2019.00840

**Published:** 2019-07-16

**Authors:** Pearlin Shabna Naziz, Runima Das, Supriyo Sen

**Affiliations:** Department of Biotechnology, School of Life Sciences, Assam Don Bosco University, Sonapur, India

**Keywords:** oudh, aroma, *Aquilaria*, perfume, methyl jasmonate

## Abstract

Agarwood (*Aquilaria* spp.) fragrance and its origin in stress make it probably the most suitable model to study stress-induced aroma. Production being confined only to certain small pockets of South and Southeast Asia, agarwood is arguably the costliest wood in the world. Formation of fragrant agarwood resin is the outcome of complex biotic, abiotic, and physical stress on the *Aquilaria* trees. The intricate mechanism by which some 150 odd fragrant molecules that constitute agarwood aroma is formed is still not clearly understood. The present review therefore aims to bring to focus this less known but highly valuable stress-induced aroma from Asia. Discussions on agarwood species, occurrence, distribution, formation, and products have been included as foundation. Although global trade in agarwood and its products is estimated at US$6 billion to US$8 billion, no reliable data are readily available in literature. Therefore, an effort has been made to review the current status of agarwood trade. The element of stress and its correlation to agarwood aroma is discussed in the subsequent sections. Natural agarwood formation as well as technologies and interventions in agarwood induction are stress-based (natural and artificial injury, insect and fungal attack, chemical induction). The molecular triggers are gradually coming to light as new studies are implicating jasmonate, LOX signaling, and other stress reaction routes as the source of agarwood aroma. This review therefore has strived to compile the information that is scattered across scientific as well as other authentic literature and update the reader on the current status. More information about the specific roles of other vital stressors like insects, abiotic, and genetic factors is eagerly awaited from ongoing and future research to further understand the unique fragrance of agarwood.

## Introduction

Agarwood is quite unusual, since stressed, diseased, and malformed trees are preferred over healthy, luxuriant ones. In fact, the infected heartwood of agar is the most expensive wood in the world. The fervor of antiquity associated with agarwood is evident from texts and traditions of the most ancient cultures. Agarwood, aloeswood, eaglewood, *agaru*, and *gaharu* are all synonyms for the resinous, fragrant, and valuable heartwood of mostly *Aquilaria* spp. belonging to the family Thymelaeaceae. Owing to their widespread use in medicinal, aromatic, and religious purposes, agarwood is also known as the *Wood of the Gods*. Trade in agarwood is over 2,000 years old with consumer centers located mostly in the Middle East and Eastern Asia, while the supply came through traditional routes from agarwood growing zones that range from South Asia (that includes China, Northeast India, and Bangladesh) through to continental Southeast Asia and to the Indo-Malaysian archipelago (Hou and Van Steenis, 1960). After the 1970s, there has been a phenomenal increase in the demand for agarwood particularly from the Middle East. Since most of the agarwood trees grow in wild areas, concern over their sustainable utilization is justified. Due to the rampant destruction of natural habitats, most agarwood-bearing species have been relegated to the status of endangered species. In fact, all 19 known *Aquilaria* species are included under CITES (CITES, UNEP-WCMC Species Database: CITES-Listed Species, 2019) and the Red List of the IUCN (IUCN Red List of Threatened Species, 2019). The trade in agarwood is largely unorganized, and often fake and adulterated wood is pushed into markets as cheaper agarwood. Even under such circumstances, the price per kilogram of agarwood can range from US$100 to US$100,000 depending on the quality of the material. In the market, agarwood is available in various grades depending on the resin content, specific gravity, color, and sometimes odor. For instance, in Malaysia, *Kalambak* and *Gaharu* are the two popular grades of agarwood, while in Japan, *Kanankoh* (or *Yara*) and *Jinkoh* stand for the highest- and lowest-quality agarwood, respectively. Similarly, in India, four wood types, *viz*., True agar (black), *Bantang* (brown), *Butha* (mixture of agarwood with non-agar), and *Dhum* (yellow), are commercially marketable grades of agarwood, based on the decreasing intensity of dark coloration caused by resinous deposits (Naef, 2011). Formation of agarwood occurs by an intricately orchestrated stress response mechanism caused by injury due to physical, microbial or entomological activity within the wood. Secondary compounds rich in oleoresins are formed as a result of the stress and get deposited in the heartwood that turns dark and heavier (Ng et al., 1997). However, the frequency of natural infection is low and is rather a matter of chance, as seen in case of plantations where only 7–10% of the trees ultimately form resin. In fact, the phenomenon of natural agarwood formation is yet to be properly understood, and the need for proper scientific inquiry and evolution of technologies adoptable for sustainable production of agarwood is a matter of urgent concern. The present review is an attempt at analyzing this unique phenomenon of stress-induced formation of agarwood. By critically analyzing the different aspects of agarwood formation, its chemical diversity, and commercialized products, the influence of stress upon aroma comes across as a prominent inducing factor. Moreover, biotic (insect, fungus) interactions established over thousands of years of co-evolution have led to a deeper scientific interest into the origins of this unique aroma. Agarwood, therefore, can arguably be the best example of stress-induced aroma. Therefore, a review that catalogues, deliberates, promotes, and articulates the information scattered in scientific literature and other sources can provide the basis for a mechanistic understanding of such phenomena, as well as to initiate and regulate future research in this exciting field.

## Agarwood—Products, Species, and Molecules

**Figure 1:**
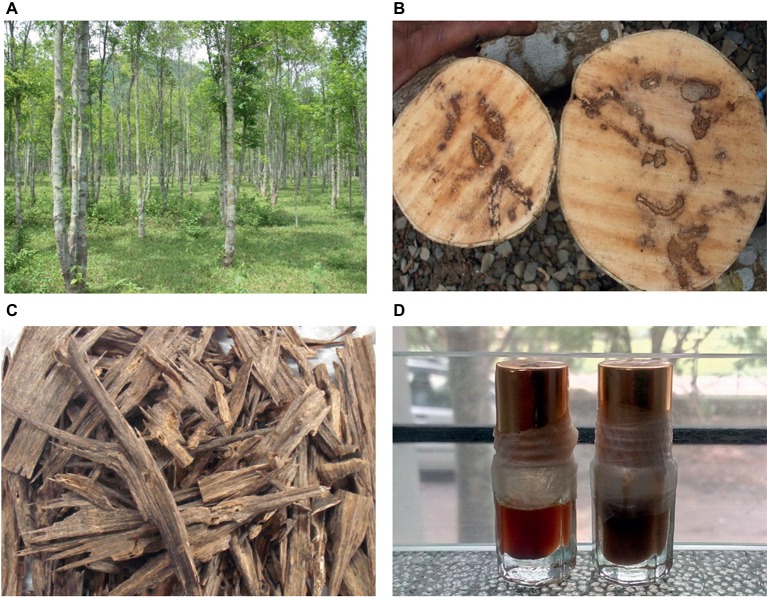
Agarwood and its products: *Aquilaria* trees growing in a plantation located in Assam, India **(A)**; resin impregnated stems are seen **(B)**, which, when chiseled and cleaned, reveal the resinous portions **(C)**; the wood is distilled to produce the fragrant agarwood oils **(D)**.

Ancient Egyptians are believed to be the first users of agarwood in death rituals more than 3,000 years ago, and the trade of perfume products, including agarwood, flourished through the trade routes of antiquity. Presently, agarwood oil is used in perfumes and cosmetic products and medicines throughout the world. Agarwood chip is made into incense, or the wood is carved into artistic shapes, which are in very high demand in the international market. Agarwood oil is by far the most precious essential oil in the world, with prices reaching as much as US$50,000 to US$80,000 per liter (Persoon, 2007). The foremost use of agarwood products is in the flavor and fragrance industry. Most of the international trade in agarwood is for production of perfume that is used for aesthetic and religious purposes. In the Middle East, agarwood is used as *agar attar* and incense and the oil also serves as the base note for perfumes and essential oil formulations. Agarwood is also used in aromatherapy and medicine. In Chinese *Materia medica*, *Aquilaria-*derived therapies for abdominal pain, vomiting, diarrhea, and asthma are recorded. In Ayurveda, formulations derived from agarwood are prescribed as carminative and refrigerant, while in Unani, it is used as a stimulant, stomachic, laxative, and aphrodisiac. Pharmacognosical studies on agarwood have shown anti-cancer, analgesic, anti-inflammatory, and anti-depressant properties (Gunasekera et al., 1981; Okugawa et al., 1993; Zhou et al., 2008). The process for the extraction of the oil is by traditional hydrodistillation where sun-dried chips with dark resin are soaked in water for 2–3 months and thereafter boiled to recover the oil. Assam in the Northeast of India is one of the globally well-known centers of agarwood where more than 10,000 distillation units are functioning, providing livelihood to around 200,000 people. The reader is referred to Figure 1 and also to one of our earlier publications (Sen et al., 2015) for a detailed understanding of the agarwood production process and products.

*A. malaccensis* and *A. crassna* are the best-known species that produce agarwood. *Aquilaria* is derived from the Latin word *aquila* meaning eagle. Trees of other genera, namely, *Gonystylus* and *Gyrinops*, also produce agarwood but are not recognized for production at commercial scales. Four species, *viz., A. malaccensis, A. crassna, A. sinensis*, and *A. filaria*, are commercially exploited for agarwood production (Barden et al., 2000). It is most notable that agarwood production is confined to a very specific geographical location within South and Southeast Asia, involving only about 15 mostly smaller countries. Even among the larger nations such as India and China, only in particular regions is agarwood grown. For *A. malaccensis*, 10 countries have been identified as range states, which include Bangladesh, Bhutan, India, Indonesia, Iran, Malaysia, Myanmar, Philippines, Singapore, and Thailand. However, since no evidence of the species was found in Iran, it was later excluded from the list (Oldfield et al., 1998). *Aquilaria* spp. can survive on most types of soil and grow between altitudes of up to 1,000 m in locations with an average temperature of 20–22°C. *A. malaccensis* can grow up to a height of 40 m with 60 cm stem diameter, but only 10% of mature trees form agarwood (La Frankie, 1994). Age is a major factor as infected trees produce resin from the age of 15 years, and trees aged 50 years and more produce the best yields of agarwood (Chakrabarty et al., 1994). The global demand for agarwood therefore threatens to outstrip the supply from the limited geographical zones where production is confined to. The desire for high monetary gains from agarwood trade has led to unscrupulous collection from wild habitats. Appendix II of the Convention on International Trade in Endangered Species of Wild Fauna and Flora (CITES) includes all known *Aquilaria* species, which places major restrictions on trade of agarwood tissue and raw materials (CITES, 2019). The IUCN Red List of Threatened Species includes the 19 *Aquilaria* species under different categories. *A. malaccensis, A. rostrata, A. khasiana*, and *A. crassna* are listed under “Critically endangered” category while eight other species, *viz., A. banaensis, A. beccariana, A. cumingiana, A. hirta, A. filaria, A. rugosa, A. yunnanensis*, and *A. sinensis* are listed as “Vulnerable.” Another seven species, *A. parvifolia, A. brachyantha, A. citrinicarpa, A. apiculata, A. subintegra, A. baillonii*, and *A. urdanetensis* are cited as “Data deficient” in the list (IUCN, 2019).

## Global Trade in Agarwood—Current Status

Trade in agarwood and its products dates back to ancient times and some texts suggest that the famous Silk Route was used by traders to carry agarwood from China to the Middle East often *via* India. Traditionally the production is from South and Southeast Asia (India, Bangladesh, China, Malaysia, Thailand, Indonesia, and Vietnam), and the markets are primarily in the Middle East (Saudi Arabia, Kuwait, UAE) as well as Far East (Japan). However, presently, there has been an expansion with emerging centers of supply (Australia, Sri Lanka, Lao PDR, Papua New Guinea) and demand (France, Italy, other European countries).

**Figure 2:**
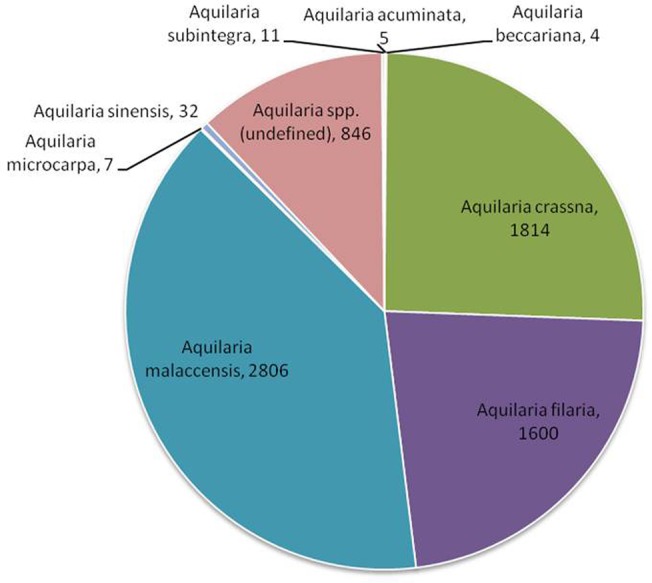
Shares of different Aquilaria species in global trade [values indicate number of times transactions (both export and import) occurred during 2008–2018]. (Source of data: CITES Trade Database, 2019).

**Figure 3:**
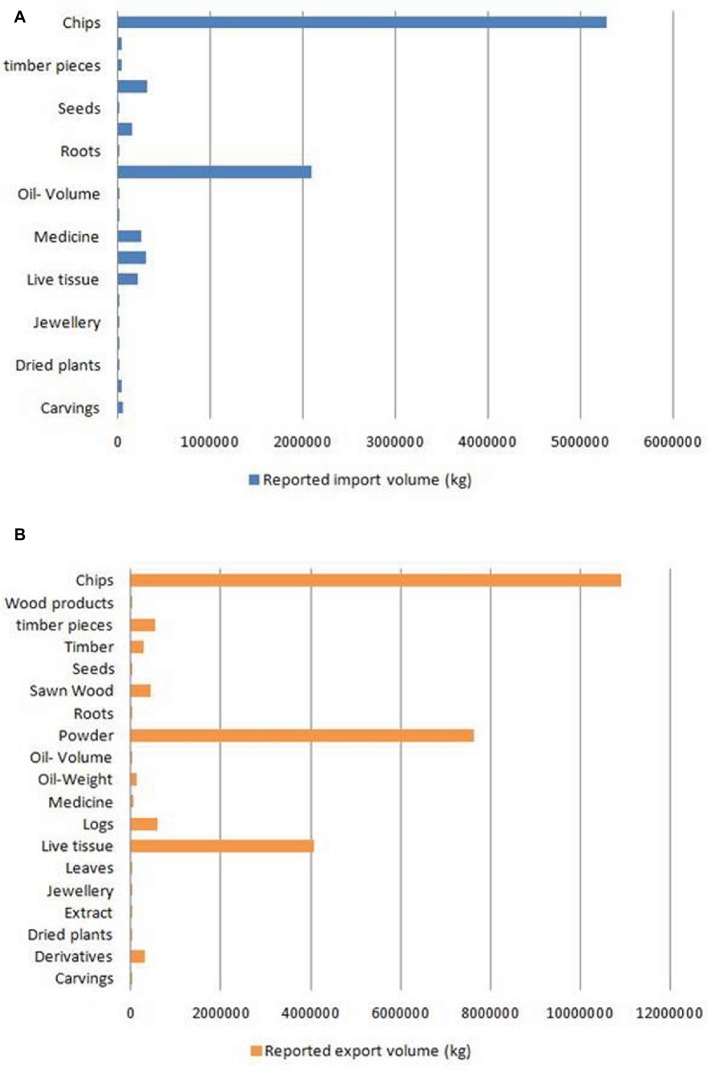
Reported import **(A)** and export **(B)** volumes of different types of agarwood-based products in the global market during 2008–2018. (Source of data: CITES Trade Database).

**Figure 4:**
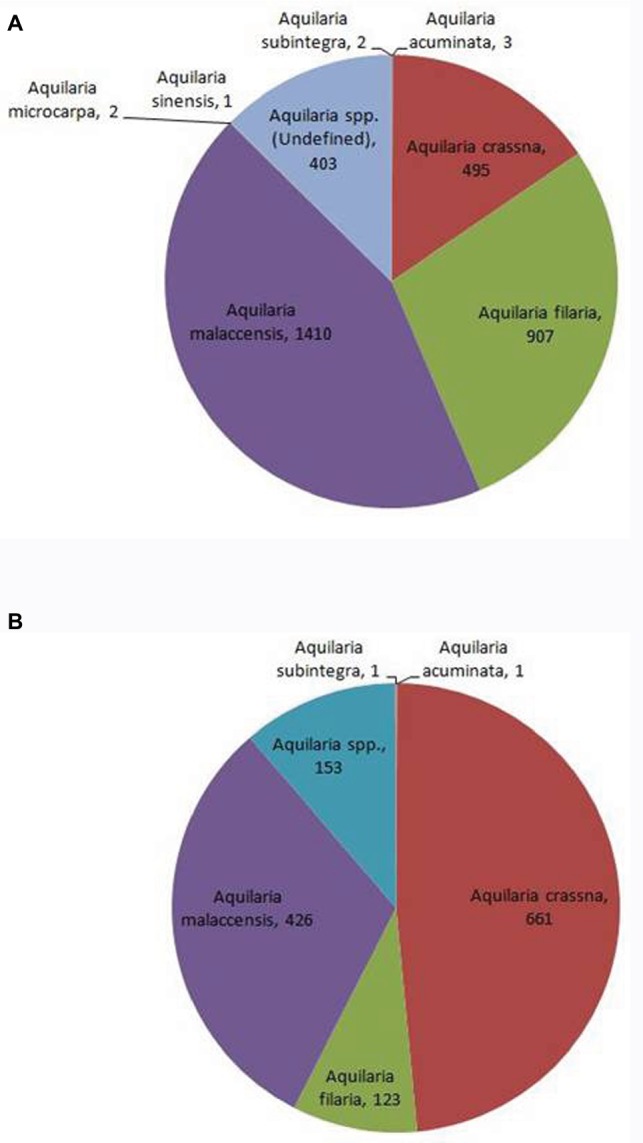
Shares of different agarwood species in global trade of agarwood chips **(A)** and oil **(B)** during 2008–2010. The values indicate number of times transactions were recorded for the species. (Source of data: CITES Trade Database, 2019).

With global market estimated at US$6 billion to US$8 billion (Akter et al., 2013), agarwood is among the most commercially valuable plant species in the world. However, due to an undefined market and clandestine trade practices, authentic data on the market are unavailable. CITES, which monitors global trade in endangered species, has ranked *Aquilaria* among the most traded plant species globally^1^. The CITES Trade Database (2019)^2^ is regarded as an authentic source of data on the global trade in wild species. Data on global trade in agarwood for last 10 years (2008–2018) was downloaded from the website and analyzed. The data revealed that *A. malaccensis* followed by *A. crassna* and *A. filaria* are the agarwood species that have dominated the international trade for the past 10 years (Figure 2). Among the countries involved in trade of agarwood products, Saudi Arabia followed by Singapore and Kuwait were the top importers of agarwood and its products. Among the exporters of agarwood, Singapore, Thailand, UAE, Indonesia, and Malaysia are at the top positions. It should be noted that countries such as Singapore and UAE are important trading but not production centers like Thailand or Malaysia. Different types of products ranging from agarwood/timber logs to ornaments made of resinous wood are traded. Among the product types, agarwood chips followed by powdered wood constitute the majority of trade with regard to both import and export (Figure 3). Other commonly traded products include timber and timber pieces, live tissue, sawn wood, medicinal preparations, and other derivatives. Agarwood chips are the most popular traded item, the majority of which is sourced from *A. malaccensis*, followed by *A. filaria* and *A. crassna* (Figure 4A). Data show that a total of 5,182,420 kg of agarwood chips was imported during 2008–2018 mostly into Saudi Arabia and Singapore. During the same period, a total amount of 10,902,850 kg of chips was exported, mostly by Indonesia, Thailand, Malaysia, and Bangladesh. An important agarwood product is the essential oil. However, because the comparative volume of agarwood essential oil traded (in kilograms or liters) is understandably much smaller compared to chips or timber/logs, it does not figure prominently in Figure 3. The data on agarwood oil were therefore analyzed separately and revealed that *A. crassna* followed by *A. malaccensis* and *A. filaria* are the major species used for oil production during the last 10 years (Figure 4B). Since data on agarwood oil trade were available both in weight (g/kg) and in volume (liter) terms, the analysis of the data was made individually. It revealed that during 2008–2018, a total of 18,775 kg and 1854 L of oil were imported mainly into Saudi Arabia, Singapore, and UAE. At the same period, a total of 124,680 kg and 7,121 L of oil were reported to have been exported mainly by countries like Vietnam and UAE.

Apart from *Aquilaria, Gyrinops* is another genus in the family Thymeliaceae that produces fragrant resin. During 2011–2015, there was a higher and sustained growth in the trade of *Gyrinops* wood chips as compared to *Aquilaria* (Cites Trade Data Dashboard: http://cites-dashboards.unep-wcmc.org). The species *G. versteegii, G. caudata, G. ledermanii*, and *G. walla* were traded with a total reported volume of 148,885 kg in imports and 181,600 kg in exports globally predominantly of chips^3^. There is a steady growth in trade, and countries like Sri Lanka and Papua New Guinea are emerging as new centers of agarwood export.

## Agarwood Formation—Mechanisms and Interventions

The fragrant heartwood of agarwood is formed by stress, leading to an accumulation of a variety of secondary metabolites, mostly terpenoids. Universally, terpenoids or isoprenoids are derived from the condensation of the two five-carbon units, isopentenyl diphosphate (IPP) and dimethylallyl diphosphate (DMAPP). The mevalonate (MVA) and the 2-C-methyl-D-erythritol 4-phosphate (MEP) pathways are known as the two universal routes for biosynthesis of terpenoids. However, these two pathways are not ubiquitously distributed in all organisms. The MVA pathway is found in fungi and animals and in the cytosol of plants, while the MEP pathway is used by bacteria and in plant plastids. The condensation of IPP and DMAPP is catalyzed by prenyltransferases, which produce the linear prenyl diphosphate precursor for each class of terpenes: geranyl diphosphate (GPP), farnesyl diphosphate (FPP), and geranylgeranyl diphosphate (GGPP) for the mono-, sesqui-, and diterpenes, respectively. Terpene synthases are a very large enzyme family that play a key role in terpene biosynthesis, since they catalyze the cyclization of GPP, FPP, and GGPP to form the carbon skeletons of the terpenes, and are thus at the origin of the extremely wide diversity of final structures (Ashour et al., 2010). Agarwood is composed of a wide variety of compounds that impart aromatic and medicinal properties. More than 150 compounds have been identified so far, mostly sesquiterpenoids, chromones, and volatile aromatic compounds. A detailed compilation of the volatile and semi-volatile constituents of agarwood, from *Aquilaria* species, mainly *A. malaccensis, A. sinensis*, and *A. crassna*, has been presented in a comprehensive review of agarwood compounds (Naef, 2011). The most prominent groups of compounds include agarofurans, cadinanes, eudesmanes (and selinanes), valencanes (and eremophilanes), guaianes, prezizanes, vetispiranes, 2-(2-phenylethyl)-chromones, and other common volatile aromatics such as benzene, toluene, and naphthalene.

Agarwood is formed irregularly in patches on the stems of *Aquilaria* spp. The fragrant oleoresinous heartwood is formed by the activation of secondary metabolite biosynthesis pathways as a defense mechanism. Healthy *Aquilaria* trees do not produce these fragrant compounds, and the formation of agarwood has invariably been found to be associated with mechanical injury and with attack by insects and microbes in plants that have attained a considerable age. Plants synthesize and accumulate secondary metabolites in response to stress caused by injury and infections. The association of fungi with formation of agarwood is well established. In the earliest study by Bose (1938), a fungus from the diseased wood of *A. malaccensis* was isolated, which was designated as a member of the fungi imperfecti. This was followed by isolation of *Epicoccum granulatum* from *A. malaccensis*, which was again artificially re-inoculated into the wood and re-isolated (Bhattacharya et al., 1952). Fungal association in *A. malaccensis* was also established by the isolation of *Cytosphaera mangiferae* in Bangladesh (Jalaluddin, 1970). The role of mechanical injury has been emphasized by some workers, suggesting that there was no primary role of fungus in the formation of agarwood (Gibson, 1977; Rahman and Basak, 1980), but was formed in response to stress-induced injuries, which were later colonized by other organisms (Chalermpongse et al., 1990). It was also believed that insect infestation by taking advantage of the cracks and crevices along the bark creates injuries, which are then colonized by fungal species, thereby representing a route to agarwood formation. One such example is the stem borer *Zeuzera conferta*, whose larvae enter the *Aquilaria* stem through cracks and crevices and tunnels inside as it feeds on the wood. This movement creates injury and facilitates fungal infection at the injured portions of the tree. Various mechanical injury methods are adopted in the field to invite the causal agents for the formation of agarwood. In one such study by Pojanagaroon and Kaewrak (2005), different techniques of injury for the formation of agarwood in *A. crassna* were compared. The results indicated that holes made with screws, wounds inflicted with chisels, and bark removed with hatchets gave dark yellow-brown to dark discoloration, while nails hammered into the trunk gave dark brown to black, and hammers beaten on the trunk gave only little discoloration.

The role of fungus-plant association in agarwood formation is beginning to get clear. Fungal diversity in the infected portions of *A. malaccensis* trees growing in natural forests of West Malaysia was estimated by adoption of PCR amplification of the internal transcribed spacer (ITS) region, apart from culture morphology and microscopic studies (Mohamed et al., 2010). In the study, conventional culture methodology revealed *Cunninghamella, Curvularia, Fusarium*, and *Trichoderma* species as members of the agarwood community. In the wood samples analyzed by PCR, *Lasiodiplodia* species were identified. Neighbor-joining trees indicated the presence of five distinct taxa, indicating a complex community that might be involved in agarwood formation. In another study (Cui et al., 2011), the fungal diversity from the infected stem tissues of *A. sinensis* was also determined by an ITS-rDNA approach. Isolates were grouped into 14 genera, of which *Fusarium* and *Phaeoacremonium* were the most dominant ones. The artificial inoculation with *Fusarium solani, F. sambunicum*, and *F. tricinctum* into *A. microcarpa*, sourced from different agarwood growing regions of Indonesia, revealed inoculation of Gorontalo-originated *Fusarium* into *A. microcarpa* stems caused the largest and fastest infection as compared to *Fusarium* originating from West Sumatra, West Kalimantan, or Jambi within 2–6 months (Santoso et al., 2011). However, in the report, the success of infection was determined based solely on the size of infection, which limits the scope of this study. They subsequently found that a negative correlation exists between the formation of agarwood and total phenolics. In subsequent studies, a novel method called the Whole-tree agarwood-inducing technique (Agar-Wit) has been reported (Liu et al., 2013). In this method, chemical inducers of agarwood are injected into xylem vessels of agarwood trees that lead to formation of resin deposits in the entire tree. The authors have claimed a 28 times higher yield over conventional methods of induction such as physical injury and fungal inoculation. In a recent study by Chen et al. (2018), a fungus, *Rigidoporus vinctus*, was isolated from the inner layer of infected *A. sinensis* trees. When the fermentation liquid of the fungi was inoculated into an *A. sinensis* tree, agarwood was found to be induced. In addition, a novel method called trunk surface agarwood-inducing technique (Agar-Sit) was developed to produce agarwood with *R. vinctus*. According to the results, when the combination of Agar-Sit and *R. vinctus* was used, agarwood could be induced with higher yield and better quality of resin (Chen et al., 2018). Another method has been developed for large-scale production of agarwood, where essentially a combined approach is used, based on physical wounding and chemical induction. Here, the inducing agent is injected into the *Aquilaria* tree *via* an aeration tool inserted into the wound (Blanchette and Heuveling, 2009). This method is now known as cultivated agarwood kit (CA-kit). Similarly, another new technique for agarwood induction is known as biologically agarwood-inducing technique (Agar-Bit), which yields better quality and quantity of agarwood. It was found that the Agar-Bit method upregulated the expression of some biological synthase genes (farnesyldiphosphate synthase, sesquiterpene synthase, and chalcone synthase) compared to the mechanically stimulated agarwood (Wu et al., 2017; Tan et al., 2019). Subsequently, Subasinghe et al. (2018) carried out an artificial induction by *Aspergillus niger* and *F. solani* for agarwood resin formation in *Gyrinops walla* trees. Those fungal species were mostly found in naturally formed resinous *G. walla*. GC-MS analysis showed that jinkohol, agarospirol, and 2(2-phenyl) chromone derivatives were present in all discolored tissues collected at 10 cm intervals of the trees inoculated with each fungus. β-Seline, γ-eudesmol, and valerenal were found in 9 out of 10 sample points in the stem.

## Terpenoid Biosynthesis

The MVA (mevalonic acid) and MEP (methylerythritol phosphate) pathways that are responsible for the production of secondary compounds of the terpenoid family have been extensively studied. The characterization of genes and enzymes involved in the pathway has further elucidated the biochemical process, thereby providing valuable information for the metabolic engineering of terpene biosynthesis. Genes coding for terpene synthases that help in the formation of the main compounds are crucial and are present in large numbers in the living world. Thousands of sequences for plant and microbial terpene synthases are deposited in public databases and putative functions have been assigned to several hundreds of them (Bohlmann et al., 1998; Davis and Croteau, 2000). A large number of genes involved in terpenoid biosynthesis have also been characterized in different plant species. In plants, metabolic engineering of the terpenoid biosynthetic pathway is more complex as the MVA pathway leads to formation of sesquiterpenes in the cytoplasm and the MEP pathway leads to formation of monoterpenes and diterpenes in the plastids. In a study conducted by Liu et al. (2015), 1-deoxy-D-xylulose 5-phosphate reductoisomerase (DXR), a key enzyme of the MEP pathway of sesquiterpene biosynthesis, has been isolated from the stem of *A. sinensis* and named *AsDXR*. Tissue expression pattern analysis showed that expression of *AsDXR* is strong in root and stem, but weak in leaf. It has also been reported that in response to methyl jasmonate treatment, *AsDXR* transcripts increased up to 34-fold as compared to the untreated control. After methyl jasmonate treatment, the level of expression of related genes including *AsDXR* increased. As a result, the sesquiterpene level also increased with the upregulation of *AsDXR* transcripts, which suggest that methyl jasmonate could induce the agarwood sesquiterpene accumulation. Genetic engineering of plants has been used to produce terpenes by successfully expressing heterologous terpene synthase genes to produce novel monoterpenes, sesquiterpenes, and diterpenes (Besumbes et al., 2004; Lucker et al., 2004; Wu et al., 2006). In one of the cited examples, Wu et al. (2006) used the patchoulol synthase gene from patchouli to transform tobacco. The resultant sesquiterpene profile showed striking similarity to patchouli oil. In the same paper, co-expression of terpene synthase genes with a gene encoding avian FPP synthase, either for cytosolic or plastidial localization, and manipulating the sub-cellular compartmentation of terpene synthases led to an increased accumulation of terpenoids in transgenic plants. The use of appropriate promoters for tissue-specific accumulation of terpenoids such as in leaves of high biomass yielding species is another possible means for enhanced recovery. In leaves, volatile and hydrophobic compounds usually accumulate in structures such as trichomes. Trichome-specific promoters can be used to drive the accumulation of the terpenes specifically into leaf trichomes, which can confer ease of recovery of the compounds (Kim et al., 2008; Shangguan et al., 2008). Sesquiterpene synthases are major enzymes for biosynthesis of sesquiterpene compounds and are important for agarwood formation. The *As-sesTPS* gene encoding a novel sesquiterpene synthase was expressed in *Escherichia coli* strain BL21 (DE3) as an inclusion body and purified by affinity chromatography. Amino acid sequencing results showed that the 27.2 kDa recombinant protein was a truncated sesquiterpene synthase from chemically induced *A. sinensis*. After refolding, the truncated As-SesTPS protein catalyzed the conversion of farnesyl diphosphate (FPP) to nerolidol, which is an important compound of agarwood. The results from qPCR and iTRAQ studied showed a higher expression level of the *As-SesTPS* gene in resinous agarwood. From this study, the mechanism of agarwood formation in *A. sinensis* and the ability of the novel gene in enhancing the quality of artificial agarwood could be partly understood. In a recent study, cloning of two new genes, *AmSesTPS1* and *AmGuaiS1*, from *A. malaccensis* has been reported (Azzarina et al., 2016). Sequence alignment showed that *AmSesTPS1* shared 99 to 100% identity with sesquiterpene synthase from *A. sinensis* while *AmGuaiS1* shared 95–99% identity with δ-guaiene synthases from *A. crassna* and *A. sinensis*. The genes were functionally characterized using two types of wood samples, i.e., wounded area (S1) and 5 cm below the wounded area (S2). The differential expression of both the genes indicated that they were upregulated in the distal area from the wound, and they could be re-induced later if secondary triggers such as natural biological infestation were applied. In a recent study, it has been reported that jasmonate induces expression of sesquiterpene synthase *ASS1*, which is a major enzyme in the biosynthesis of agarwood sesquiterpenes in *A. sinensis* (Xu et al., 2017). A transcription factor, AsMYC2, has been characterized as an activator of *ASS1* expression. The results suggest that AsMYC2 participates in the regulation of agarwood sesquiterpene biosynthesis in *A. sinensis* by controlling the expression of *ASS1 via* the jasmonate signaling pathway. In a more recent study, it has been observed that hydrogen peroxide (H_2_O_2_) plays an important role in the upregulation of sesquiterpene synthase in *A. sinensis* which is an important enzyme in sesquiterpene biosynthesis (Lv et al., 2019). H_2_O_2_ induces sesquiterpene production in *A. sinensis*, increasing the expression of *AsTPS10, AsTPS16*, and *AsTPS19* genes, which are responsible for the biosynthesis of sesquiterpenes in the early stage of response to wound stress. At the same time, the presence of H_2_O_2_ also increases the accumulation of jasmonic acid (JA) and salicylic acid in *A sinensis* by activating the expression of lipoxygenase (*AsLOX*), allene oxide cyclase (*AsAOC*), allene oxide synthase (*AsAOS*), and isochorismate synthase (*AsICS*) genes.

## From Stress to Aroma: The Agarwood Model

The aroma of agarwood is an outcome of different stressors, which depend on the growth stages of the plant, its genetic predisposition, and its real-time interaction with its biotic and abiotic environment. Studies have been carried out to understand it mostly by simulating the stressful conditions in the laboratory as well as in the field. *In vitro* culture studies started revealing the mechanistic features of the terpenoid formation in response to stress. Success in establishment of cell culture system for *Aquilaria* was further exploited for the characterization of sesquiterpene synthase genes of *Aquilaria* spp. In a study by Kumeta and Ito (2010), suspension cultured cells of *A. crassna* were found to accumulate agarwood sesquiterpenes (α-guaiene, α-humulene, and δ-guaiene) upon induction by methyl jasmonate. A cDNA library made from RNA isolated from elicitor-treated suspension cultured cells was screened to identify the clones that triggered enzymatic reactions when the terpene precursor farnesyl diphosphate was fed in the media. Results showed formation of sesquiterpenes such as δ-guaiene. The genes and their encoded enzymes were the first sesquiterpene synthases to be reported. In a subsequent study, Kumeta and Ito (2010) further enhanced the understanding of agarwood biosynthetic machinery by revealing the genomic organization of the δ-guaiene synthase gene in *A. crassna*. Cloning and sequencing revealed five types of sequences in putative δ-guaiene synthases sharing more than 96% identity in exon regions, and these enzymes belonged to the class III TPS (terpene synthase genes) subfamily (Kumeta and Ito, 2011). Furthermore, Southern blotting revealed that at least five copies of δ-guaiene synthase exist in *A. crassna*. The hybridization of digested DNA of *A. crassna* and *A. sinensis* with probes made from a δ-guaiene synthase cDNA fragment resulted in different banding patterns for these two species, which can be employed in differentiating the species of *Aquilaria* by restriction fragment length polymorphism analyses, using δ-guaiene synthase cDNA probes. In a study by Kenmotsu et al. (2011), it was found that the biosynthesis of spirovetivane-type sesquiterpene in *A. microcarpa* is triggered by the treatment with methyl jasmonate due to enhanced expression of farnesyl diphosphate synthase, calmodulin, and Rac/Rop GTPase genes. Again, the genetic transformation of *A. microcarpa* with genes encoding GTPase or CAM showed that expression levels were enhanced even in the absence of methyl jamonate, suggesting that these genes play important roles in sesquiterpene biosynthesis. Tubulin, ribosomal protein, and glyceraldehyde 3-phosphate dehydrogenase genes were identified as the most stable reference genes for quantification of target gene expression in *A. sinensis* in the future (Gao et al., 2012a). MicroRNAs (miRNAs) that are important determinants in gene expression regulation during stress response and metabolic processes might be involved in agarwood formation. Gao et al. (2012b) discovered 27 novel miRNAs in *A. sinensis*, and expression levels of 10 stress-responsive miRNAs were correlated with wounding. Eight of them were wound-responsive, which shows that the existence of miRNAs is indicative of their critical role in stress reactions, leading to agar formation (Gao et al., 2012b). Similarly, for the first time, coronatine-insensitive protein 1 COI1 gene (*AsCOI1*) from *A. sinensis* has been cloned and characterized as a receptor in the jasmonate signaling pathway (Liao et al., 2015a). AsCOI1 is a conserved protein, containing F-box and LRR domains. It is mainly expressed in roots and stems, and its expression is lowest in leaves. The expression of *AsCOI1* could be significantly induced by methyl jasmonate, mechanical wounding, and heat stress *in A. sinensis* callus. It is an early wound-responsive gene that might play some significant role in agarwood formation. In another study, a lipoxygenase gene (*AsLOX1*) in *A. sinensis*, also an early wound-responsive gene, was examined; the enzyme plays a significant role in plant defense and also as a major player in the octadecanoid pathway and jasmonate synthesis (Liao et al., 2015b). Based on the transcriptome data, a full-length cDNA sequence of the LOX gene (*AsLOX1*) was cloned from *A. sinensis* by RT-PCR and RACE. The deduced amino acid sequence of *AsLOX1* has nearly 80% identity with LOX proteins of several other species, which suggests that *LOX* belongs to the class of conserved proteins. The phylogenetic tree analysis reveals that *AsLOX1* is more closely related to *Theobroma cacao LOX* than to other plants. *AsLOX1* expression in tissues follows the pattern already observed with *AsCOI1*. In both the methyl jasmonate and crush wounding treatments, the expression of *AsLOX1* was significantly higher in *A. sinensis* calli. From these observations, it can be concluded that the *AsLOX1* gene belongs to early damage-responsive genes, and its expression increases in response to methyl jasmonate stress and wounding, indicating its role in defense responses leading to wounding-induced agarwood formation. In a recent study, it has been demonstrated that heat shock can up-regulate the expression of the genes *LOX, AOS*, and *AOC* involved in jasmonate signaling and hence the accumulation of agarwood sesquiterpenes in *A. sinensis* cell suspension cultures. The total sesquiterpene content in the heat shock samples increased by 9-, 15-, and 35-folds at 1, 3, and 5 days, respectively, as compared to that of the nordihydroguaiaretic acid (NDGA)-containing samples. NDGA is a selective inhibitor of jasmonate signaling that might lead to a decrease in the production of sesquiterpenes. It was observed that after 24 h of the heat shock treatment, much less volatile oil content was detected in the samples containing NDGA. In the same study, exogenously applied methyl jasmonate was found to stimulate the formation of sesquiterpene compounds in *A. sinensis* (Xu et al., 2016; Chen et al., 2017). The mechanism of fungus-induced agarwood formation in calli of *A. sinensis* by *Lasiodiplodia theobromae* was investigated by Han et al. in 2014 (Han et al., 2014). They detected JA in the fermentation liquor of *L. theobromae*, which stimulated the biosynthesis of important agarwood sesquiterpenes such as α-guaiene, α-humulene, and δ-guaiene. Subsequently, in a recent study, Chini et al. (2018) isolated JA, its methyl ester, and three furanonyl esters from the grapevine pathogen *L. mediterranea*. JA ester lasiojasmonate A (LasA), the first reported naturally occurring JA-furanone, reportedly activates many JA-regulated responses in *Arabidopsis thaliana*, i.e., protein degradation, gene expression, and physiological processes. These responses require LasA conversion into JA, formation of the bioactive plant hormone JA iso-leucine (JA-Ile), and its recognition by the plant JA-Ile complex. LasA occurs at late infection stages to induce plant JA responses and can facilitate fungal infection. This could be the basis for future studies on the role of fungal JAs in inducing agarwood formation. Ye et al. (2016) treated *A. sinensis* with formic acid and studied the transcriptome of different parts of the plant such as the white wood part (B1), the transition part between agarwood and white wood (W2), the agarwood part (J3), and the rotten wood part (F5). The blast result shows that the genes of five species, i.e., *Vitis vinifera, Ricinus communis, Populus trichocarpa, Glycine max*, and *Hordeum vulgare subsp. ulga*, have the highest similarity with the 53.83% annotated unigenes of *A. sinensis*. The differential expression of the transcription factors WRKY and MYC2 in different *A. sinensis* samples was also studied. The expression level of the gene encoding the MYC2 transcription factor did not show variation in *A. sinensis* samples, which suggests that MYC2 did not play a critical role in formic-acid-induced agarwood formation. In contrast, the maximum expression level for the WRKY transcription factor gene was observed in the J3 sample, decreased in the W2 sample, and dropped to the lowest level in the B1 sample. From this result, a positive association was found between the expression levels of the WRKY transcription factor and the sesquiterpenoid synthesis gene. In a subsequent study (Wang et al., 2016), it was determined that salinity stress could induce the production of 41 2-(2-phenylethyl)chromonesin *A. sinensis* calli. The primary deep-sequencing transcriptome profiling of *A. sinensis* was conducted under salt stress condition, which showed a wide variety of differentially expressed genes in response to salinity stress. From the qRT-PCR and RNA-seq analysis, it could be concluded that under salt stress condition, genes encoding the mitogen-activated protein kinase kinase kinase (MAPKKKA, MAPKKK2, and MAPKKK3), calmodulin, WRKY transcription factors (WRKY39, WRKY40 and WRKY75), caffeoyl-CoA-O-methyltransferase, and chalcone synthase 1(CHS1) were highly expressed as compared with the control *A. sinensis* calli. The plant-pathogen interaction pathway genes for stilbenoid, diarylheptanoid, and gingerol biosynthesis; plant hormone signal transduction; and phenylpropanoid biosynthesis were also induced in response to salt stress. The same group (Dong et al., 2018) also studied the effect of some other abiotic stresses such as drought, salt and cold stress, and hormones on the production of 2-(2-phenylethyl) chromones. It was found that under salt and drought stresses, there are increased production of 6,7-dimethoxy-2[2-(4′-methoxyphenyl)ethyl]chromone (AH8) and 6,7-dimethoxy-2-(2-phenylethyl) chromone (AH6) at 20 days. Ion stresses triggered by KNO_3_, CaCl_2_, and (NH4)_2_SO_4_ also considerably increased the content of AH6 and AH8, and KNO_3_ induced strongly the production of AH6 and AH8 at 20 days. Although cold treatment failed to induce chromone formation, hormonal treatment using methyl jasmonate, salicylic acid, and abscisic acid induced some amount of 2-(2-phenylethyl) chromones in *A. sinensis* calli within 20 days. In another study by Yang et al. (2016), 2-(2-phenylethyl)-chromone accumulation was found to depend on agarwood formation time. They characterized and analyzed 2-(2-phenylethyl)-chromone derivatives from *A. crassna*. A total of 56 chromones, including seven 5,6,7,8-tetrahydro-2-(2-phenylethyl)-chromones(THPECs), five 5,6-epoxy-2-(2-phenylethyl)-chromones (EPECs), seven 5,6,7,8-diepoxy-2-(2-phenylethyl) chromones (DEPECs), and 37 2-(2-phenylethyl)-chromones of the flindersia type (FTPECs), were characterized from three samples induced by artificial injury in different time frames It was observed that the relative contents of DEPECs and EPECs were down-regulated but THPECs and FTPECs were up-regulated for the samples from 2, 4, and 5 years of the agarwood formation time. The relative content of six FTPECs, *viz*., 6,8-dihydroxy-2-[2(4-methoxy)phenylethyl]chromone, 6-methoxy-7-hydroxy-2-[2-(4-methoxy)-phenylethyl]chromone, 6-hydroxy-2-(2-phenylethyl)chromone, 6,7-dimethoxy-2-(2-phenylethyl) chromone, 2-[2-(4-methoxy)phenylethyl]chromone, and 2-(2-phenylethyl)chromone, were also upregulated. In *Aquilaria* species, FTPECs are comparatively highest followed by THPECs, while EPECs and DEPECs are relatively lower. It has been reported that DEPECs and EPECs have not been found in any other plants except *Aquilaria* species (Liao et al., 2018).

Agarwood trees are naturally infested by the insect *Zeuzera conferta* leading to injuries that stimulate resin formation. However, only a few studies exist on this aspect. Kalita et al. (2015) studied a total of 3,824 *A. malaccensis* trees from Northeast India. It was found that only 7.14% of trees below 8 years were infested by the insect *Z. conferta*. The maximum infestation by the borer insect (34.13%) was seen in trees of the age group 8–16 years and in 13.40% of trees above the age of 16 years. During this study, it was also observed that in all conditions, the infection by *Z. conferta* of *A. malaccensis* varied with the type of plantation, be it “pure” or “mixed.” The infestation in the pure plantations of *A. malaccensis* was higher (25.09%) than in the mixed one with other tree species (22.38%). In a recent study of a tritrophic system, the herbivore *Heortia vitessoides*, its host plant *A. sinensis*, and its predator *Cantheconidea concinna* were included (Qiao et al., 2018). Herbivore-damaged *A. sinensis* plants released higher amounts of volatiles than undamaged and mechanically damaged plants. After 1 day of initial herbivore damage, *A. sinensis* plants released large amounts of volatile organic compounds (VOCs), which were divided into two groups, green leaf volatiles (GLVs) and terpenoids. Mostly, the amount of six GLVs [3-hexanol, (*Z*)-3-hexen-1-ol, (*E*)-2-hexen-1-ol, (*Z*)-3-hexenyl acetate, 2-hexen-1-ol, acetate, and 3-hexanone] and six terpenoids [β-myrcene, (*E*)-β-ocimene, (*Z*)*-*β-ocimene, linalool, caryophyllene, and α-farnesene] increased. Of these, (*Z*)-β-ocimene and two GLVs [(*Z*)-3-hexen-1-ol and (*Z*)-3-hexenyl acetate] were predominant in all treatments. The release of volatile compounds gradually decreased over the next 2–3 days, including three GLVs [(*Z*)-3-hexen-1-ol, (*E*)-2-hexen-1-ol, and (*Z*)-3-hexenyl acetate] and four terpenoids [(*E*)-β-ocimene, (*Z*)-β-ocimene, linalool, and α-farnesene]. However, some compounds increased specifically, one alcohol [2-decen-1-ol], three aldehydes [octanal, nonanal, and decanal], and one ketone [6-methyl-5-hepten-2-one]. In wind tunnel bioassays, mated *H. vitessoides* females showed a preference for undamaged plants over herbivore and mechanically damaged *A. sinensis* plants. In Y-tube bioassays, *C. concinna* preferred odors from herbivore-damaged plants over that released by undamaged ones, mainly after the early stages of insect attack. In a study carried out by Sen et al. (2017), a chemometric evaluation of agarwood and fungus interaction was done by chemical profiling, along with statistical analysis over three platforms, *viz.*, interaction of agarwood callus, juvenile plants, and resinous wood chips with a related *Fusarium*. In the study of callus and fungus interaction, the accumulation of aroma compounds, including pentatriacontane, 17-pentatriacontene, tetradecane, and 2-methyl, increased, and there was an activation of pathways associated with defense and secondary metabolism, thereby indicative of links to aroma production. Fungal interactions in juvenile plants and resinous wood chips indicated formation of terpenoid precursors (e.g., farnesol, geranylgeraniol acetate) and agarwood sesquiterpenes (e.g., agarospirol, γ-eudesmol). Correlation network analysis revealed the possible regulation of sesquiterpene biosynthesis in relation to that of squalene/phytosterol synthesis. Also, the contribution of fungal metabolites in the aroma (e.g., dodecane, 4-methyl, tetracosane) was indicated. In a recent study, differentially expressed proteins related to a wound response were examined in *A. malaccensis* (Lee et al., 2018). Proteins were extracted from *A. malaccensis* tree stems at 0, 6, 12, and 24 h after wounding separated by 2D electrophoresis and sequenced and identified using MALDI-TOF-MS. Two proteins predicted as malate synthase (MS) and nicotinamide adenine dinucleotide phosphate (NADPH) quinone oxidoreductase subunit 2B were found, which showed differences in expression due to wounding. Both the proteins were directly or indirectly related to wound in plants and may be functional in the mechanism of agarwood formation.

## Conclusion

**Figure 5:**
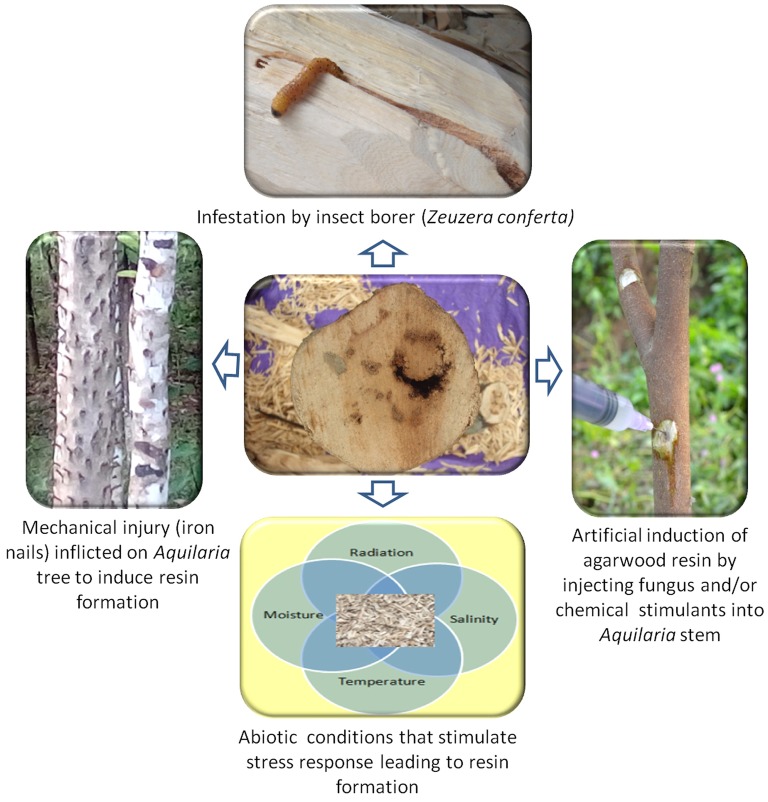
The role of stress in agarwood aroma: Multiple sources of stress in *Aquilaria* species trigger a complex response that leads ultimately to the formation of fragrant resins.

Fragrant agarwood formation is an excellent example of natural value addition due to stress. However, the scientific understanding of the exact mechanism of stress-induced agarwood aroma formation is still unclear. Further, the low frequency of natural agarwood formation has skewed the demand/supply ratio, resulting in overexploitation and destruction of natural habitats and entry of fake woods into the market, which has become a threat to the industry as a whole. Efforts to understand this unique phenomenon and then evolving technologies for the sustainable exploitation of this valuable resource are therefore a challenge in front of the scientific community. The resin-impregnated agarwood tissue is a repository of valuable molecules that find extensive use in flavor and fragrance industry as well as in therapeutics. The future holds tremendous promise for research on discovery of novel phytochemicals and the application in different industries. In nature, the biotic interactions leading to injury and a counter-response to the stress within the insect-fungus-plant continuum has been the overriding explanation for the aroma of agarwood and thus has been studied in detail. The technologies available for artificial induction of resin such as fungus inoculation, chemical, and mechanical injury also have strongly derived from a stress mechanism. The aspect of abiotic stress response in agarwood is a new dimension, which is now being looked at and reports are already becoming available. Figure 5 summarizes the diversity of stressors impacting agarwood scent. Obviously, the key to understanding the scent of agarwood is to decipher the exact stress-response mechanism during different life stages and genotypes of *Aquilaria* trees while interacting with a variety of biotic and abiotic stressors. Understanding this interplay will provide newer insights and potentially exciting opportunities for improving the quality and quantity of agarwood and similar other essential-oil-bearing organisms.

## Author Contributions

SS helped in ideation and planning, supervised the drafting, wrote and corrected the document. PN and RD wrote the manuscript partly, collected material, and organized the text.

### Conflict of Interest Statement

The authors declare that the research was conducted in the absence of any commercial or financial relationships that could be construed as a potential conflict of interest.

## Footnotes

^1^http://cites-dashboards.unep-wcmc.org

^2^https://trade.cites.org

^3^https://trade.cites.org
